# The impact of an integrated PBL curriculum on clinical thinking in undergraduate medical students prior to clinical practice

**DOI:** 10.1186/s12909-023-04450-7

**Published:** 2023-06-21

**Authors:** Feng Zhou, Aiming Sang, Qing Zhou, Qing Qing Wang, Yao Fan, Songhua Ma

**Affiliations:** 1 Practice and Training Educational Office, School of Medicine, Nantong University, Nantong, 226000 China; 2 Department of Ophthalmology, Affiliated Hospital of Nantong University, Nantong, 226001 China; 3 Education and Training Department, Affiliated Hospital of Nantong University, Nantong, 226001 China; 4 Department of Thyroid and Breast Surgery, PBL Medical Integrated Education Research Office, Affiliated Hospital of Nantong University, Nantong, 226001 China; 5 Teaching Management Office, School of Medicine, Nantong University, Nantong, 226000 China; 6 Department of Physiology, PBL Medical Integrated Education Research Office, School of Medicine, Nantong University, Nantong, 226000 China

**Keywords:** Problem-based learning, Clinical thinking, Medical education, Undergraduate medical students

## Abstract

**Background:**

Problem-based learning (PBL) is a widely adopted educational approach in medical education that aims to promote critical thinking and problem-solving in authentic learning situations. However, the impact of PBL educational mode on undergraduate medical students’ clinical thinking ability has been limitedly investigated. This study aimed to assess the influence of an integrated PBL curriculum on clinical thinking ability of medical students prior to clinical practice.

**Methods:**

Two hundred and sixty-seven third-year undergraduate medical students at Nantong University were recruited in this study and were independently assigned to either the PBL or control group. The Chinese version of the Clinical Thinking Ability Evaluation Scale was used to assess clinical thinking ability, and the students’ performance in the PBL tutorials was assessed by tutors. All participants in both groups were required to complete the pre-test and post-test questionnaires to self-report their clinical thinking ability. A paired sample t-test, independent sample t-test and one-way analysis of variance test (ANOVA) were used to compare the difference in clinical thinking scores among different groups. Multiple linear regression was conducted to analyze the influencing factors correlated with clinical thinking ability.

**Results:**

The clinical thinking ability of most third-year undergraduate medical students at Nantong University was at a high level. The PBL group had a higher proportion of students with high-level clinical thinking ability in the post-test compared to the control group. The pre-test scores of clinical thinking ability were similar between the PBL and control groups, but the post-test scores of clinical thinking ability in the PBL group were significantly higher than those in the control group. Additionally, there was a significant difference in clinical thinking ability between the pre-test and post-test in the PBL group. The post-test scores of sub-scales of critical thinking ability were significantly higher than the pre-test in the PBL group. Furthermore, the frequency of reading literature, time of PBL self-directed learning, and PBL performance score ranking were influencing factors on the clinical thinking ability of medical students in the PBL group. Moreover, there was a positive correlation between clinical thinking ability and the frequency of reading literature, as well as the scores of the PBL performance.

**Conclusions:**

The integrated PBL curriculum model has an active impact on improving undergraduate medical students' clinical thinking ability. This improvement in clinical thinking ability may be correlated with the frequency of reading literature, as well as the performance of the PBL curriculum.

## Background

Clinical thinking is an essential ability for medical staff to diagnose, analyze, and logically reason about various diseases during clinical practice [1]. It involves thinking about daily clinical problems from a logical and lateral perspective and examining them from different angles [2]. Clinical thinking is also an important educational outcome of undergraduate medical programs [3], and its cultivation is at the core of quality medical education for improving the quality of medical training. Therefore, developing clinical thinking is particularly necessary to cultivate excellent medical students.

Problem-based learning (PBL), based on constructivist theory, is a widely accepted active learning strategy in health sciences education [4, 5]. It is a problem-triggered, student-centered, and tutor-facilitated pedagogy that aims to foster active lifelong learning [6]. For medical students, PBL enables empowerment through problem-solving [7].

Despite the predominance of traditional lecture-based teaching in medical and clinical education [8], some universities, like Nantong University have incorporated an additional integrated PBL curriculum in clinical medicine teaching mode. Compared to traditional lecture teaching, PBL has been found to enhance students' interdisciplinary knowledge application, self-directed learning, critical thinking, communication, and collaboration skills [9]. However, evidence on whether the PBL curriculum contributes to the improvement of clinical thinking ability is limited.

Therefore, this study aims to investigate the impact of an additional integrated PBL curriculum on clinical thinking ability in undergraduate medical students and to identify the influencing factors of clinical thinking ability prior to clinical practice.

## Methods

### Study subjects

This study was a randomized controlled trial that involved 313 students enrolled in clinical medicine at the School of Medicine, Nantong University. Of the 313 students, 267 (85.3%) completed all the tests. These were third-year undergraduate medical students who had completed fundamental medical subjects, such as anatomy, histology, biochemistry, physiology, immunology, and pathophysiology. The participants were given the option to choose between the PBL group and the control group, and were randomly assigned to one of the two groups.

### Study design

The study was conducted over a period of five months, which corresponded to one semester. During this time, the control group participated only in the traditional lecture-based teaching program, while the PBL group took part in an additional PBL curriculum on top of the traditional teaching program. Both groups of students completed the same questionnaire test to evaluate their clinical thinking ability. To ensure data quality, the collected questionnaires were screened and any invalid ones were eliminated. The PBL group received one introductory PBL tutorial and three subsequent PBL tutorials during the semester, held in purpose-built classrooms. In contrast, the control group did not undergo any PBL process training.

### PBL curriculum design and procedures

Nantong University's medical education has been implementing a PBL model teaching reform since 2014, and an integrated PBL curriculum has been included as one of the elective courses in the professional training plan. The integrated PBL curriculum comprised three 1-h lectures on PBL ideas, mind mapping, and literature retrieval, followed by three 2-h PBL tutorial sessions and a 2-h feedback session. Each tutorial group consisted of 8–10 students, randomly assigned to each group, and facilitated by one PBL tutor. During each PBL tutorial, students and their tutor met once a week, for a total of 6 h, three times. Before each PBL case, students were unaware of the topic and contents. They were introduced to the scenario in the first class and were expected to discuss and ascertain what was known, what was unknown, what should be searched, and what should be learned. Afterwards, students searched for and learned the related information, summarizing the notes by themselves or with their team members. In the second class, students discussed and shared the information they had collected, and more scenarios were assigned by the tutors, requiring them to repeat the reflective procedures after class. In the third class, students reviewed and summarized all learning issues and the whole case with a mind map. After three panel discussions, all PBL group students had a feedback session shared by the clinician regarding the case's clinical thinking. The assessment of PBL performance was conducted by tutors at the end of every PBL case.

### Evaluation of students’ performance in the PBL tutorials

After each PBL case learning, students were assessed online by their tutors using a student performance evaluation form developed by medical education experts. The form was based on a literature review [7, 10] and the curriculum reform goal of PBL at Nantong University. Following the objectives of PBL in the school, the PBL tutorial assessment was modified to include five aspects: learning willingness and attitude, information gathering and compilation, communication and sharing skills, critical thinking and reasoning skills, and team cooperation and construction skills. Each dimension was divided into three levels: poor, moderate, and excellent. The PBL performance score depended on the level assessed by the tutor.

### Assessment of clinical thinking ability

The clinical thinking ability of the students was assessed using the Clinical Thinking Ability Evaluation Scale [11] for Medical Students. This scale consists of 24 items and three primary dimensions: critical thinking, systematic thinking, and evidence-based thinking. The questionnaire experiment included a pre-test and a post-test. The pre-test was conducted on all subjects at the beginning of the semester, while the post-test was conducted on all subjects at the end of the semester using the same questionnaire test. The data for both the pre-test and post-test were collected through the scale questionnaire.

The first part of the questionnaire collected demographic information, including age, gender, and birthplace. The second component of the questionnaire was the scale of clinical thinking ability, derived from a Chinese version of the Clinical Thinking Ability Evaluation Scale based on a consensus definition of clinical thinking ability from Delphi research [11, 12].

The Cronbach's alpha of this scale was 0.91, and the test–retest reliability was 0.84. The scale consisted of 6 items of critical thinking ability, 11 items of systematic thinking ability, and 7 items of evidence-based thinking ability, for a total of 24 items across three dimensions to report the clinical thinking ability of medical students. Participants rated each statement on a 5-point Likert scale, with 5 indicating the best response and 1 indicating the worst response. The full score was 120 points, which were converted into a hundred-point system. A clinical thinking ability score of above 60 points was considered high, a score of 40–59 points was considered general, and a score below 40 was considered poor.

### Statistical methods

Descriptive statistics were used to describe the general data of the subjects. A paired sample t-test, independent sample t-test and one-way analysis of variance test (ANOVA) were used to compare the difference of clinical thinking scores among different groups. Multiple linear regression was used to analyze the influencing factors. *P* < 0.05 was considered statistically significant. All statistical analyses were performed using IBM SPSS ver. 20.0 (IBM Corp., Armonk, NY, USA).

## Results

### Demographic variables of the two groups

Table 1 showed the demographic data of the undergraduate students who participated in this study. A total of 267 samples out of 313 students completed both the pre-test and post-test questionnaires, including 127 male participants and 140 female participants. The mean age of the samples was 21.6 ± 0.7 years. Of these, 127 students were assigned to the PBL group, and 140 participants were assigned to the control group, who did not undergo PBL procedure learning. Additionally, 45.3% of the participants came from the countryside, while 54.7% were from the city (In China, the division of “City” and “Countryside” was based on registered residence in the Birthplace category). There was no significant difference in age, gender, and birthplace between the PBL group and the control group in this study.

**Table 1:** Demographic characteristics of the participates in the sample

Item	PBL group	Control group	Statistics	*P-* value
Gender			χ2 = 64	1.000
Male	63	64		
Female	64	76		
Birthplace			χ2 = 53	0.107
City	74	72		
Countryside	53	68		
Age	21.5 ± 0.7	21.6 ± 0.5	t = -1.354	0.177

### Overall situation of clinical thinking levels of medical undergraduate students in the two groups

Table 2 presented the distribution of total scores and levels of clinical thinking in the two groups of students. In the control group, the average score was 66.2 ± 10.2 in the pre-test and 66.9 ± 11.0 in the post-test, while in the PBL group, the mean score was 65.6 ± 9.2 in the pre-test and 73.7 ± 12.6 in the post-test. Students with scores above 60 were considered to have a high level (good level plus best level) of clinical thinking. Although the percentage of high-level clinical thinking in the pre-test was similar between the two groups (82.81% vs. 81.43% for the PBL group and control group, respectively), the percentage of high-level clinical thinking in the post-test for the PBL group was significantly larger than that of the control group (92.13% vs. 85.71%). These results suggest that the PBL curriculum was effective in promoting clinical thinking ability.

**Table 2:** Descriptive analysis of the number and percentage of students according to different clinical thinking levels in the pre-test and post-test for the PBL and Control groups

Score	Clinical thinking levels	Number of students (Percentage)			
		**PBL group (*n* = 127)**		**Control group (*n* = 140)**	
		**Pre-test**	**Post-test**	**Pre-test**	**Post-test**
80–100	Best	9 (7.03%)	35 (27.56%)	12 (8.57%)	14 (10%)
60–79	Good	96 (75.78%)	82 (64.57%)	102 (72.85%)	106 (75.71%)
40–59	General	22 (17.19%)	10 (7.87%)	25 (17.86%)	19 (13.57%)
< 40	Bad	0 (0%)	0 (0%)	1 (0.71%)	1 (0.71%)

### Effect of PBL on the sub-scales of clinical thinking ability

Independent samples T-tests were carried out on the control group and the PBL group in terms of the post-test scores for the total and sub-scales of clinical thinking ability. The statistical results showed that the significance value was less than 0.05, indicating a significant difference between the two groups in terms of clinical thinking ability. Paired samples T-tests were conducted on the PBL group before and after the curriculum, and the statistical results demonstrated that the significance value was also less than 0.05, indicating a significant difference between the pre-test and post-test scores. These data suggest that PBL had a positive impact on the improvement of each dimension in clinical thinking ability (See Table 3 for details).

**Table 3:** Comparison of pre-test and post-test scores in the sub-scales and total of clinical thinking ability between the PBL and Control groups

Item	Pre-test score		Post-test score		*P* ^a^	*P* ^b^
	**PBL**	**Control**	**PBL**	**Control**		
Critical thinking ability	17.52 ± 2.35	17.8 ± 3.24	19.12 ± 3.45	17.44 ± 3.22	0.000	0.000
Systematic thinking ability	29.61 ± 4.91	29.55 ± 5.41	33.14 ± 5.95	30.16 ± 5.30	0.000	0.000
Evidence-based thinking ability	18.56 ± 2.98	18.86 ± 3.24	21.45 ± 4.02	19.36 ± 3.43	0.000	0.000
Total clinical thinking ability	65.61 ± 9.17	66.2 ± 10.25	73.71 ± 12.65	66.9 ± 11.01	0.000	0.000

^a^ A paired sample T test was used to compare the Post-test with the Pre-test scores in the PBL group. ^b^ Independent sample T test was used to compare the Post-test scores between the Control group and the PBL group

### Effect of PBL on the sub-scales of critical thinking ability

**Fig. 1:**
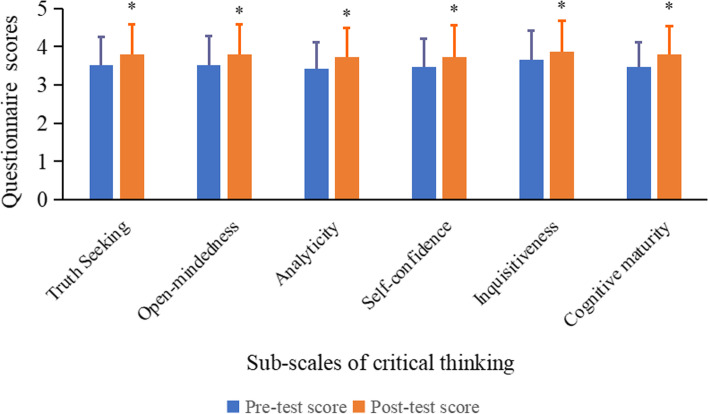
Comparison of the pre-test and post-test scores in the sub-scales of critical thinking for the PBL group. The 127 participates in the PBL group underwent a questionnaire test before and after the PBL curriculum, and the scores for the six dimensions of critical thinking were compared between the two tests. A paired sample T-test was used to compare the mean scores between the two tests, *p < 0.05, compared to the pre-test score in every dimension

In addition, we assessed the sub-scales of critical thinking ability of the 127 participants in the PBL group, and the data were shown in Fig. 1. Statistically significant differences were found in the sub-scales of critical thinking between the pre-test and post-test scores from the questionnaire testing, suggesting that the PBL curriculum effectively improved critical thinking ability.

### Influencing factors on the clinical thinking ability of medical students in the PBL group

To understand the factors influencing the clinical thinking ability of medical undergraduate students, this study focused on the PBL group of medical students and analyzed the influence of general demographic characteristics, frequency of reading medical literature, PBL performance score rankings, and time spent on PBL self-directed learning. The results are presented in Table 4, which shows the findings from independent sample T-tests and ANOVA tests that were conducted to analyze the influencing factors on clinical thinking ability. The results indicated that there was a significant difference for the factors of frequency of reading literature, time of PBL self-directed learning, and PBL performance score ranking. The higher the frequency of reading medical literature, the time of PBL self-directed learning, and PBL performance score ranking, the higher the clinical thinking ability. However, no significant difference was found for the factors of gender and birthplace on the clinical thinking ability.

**Table 4:** Analysis of influential factors on the clinical thinking ability in the PBL group

Item	n	Scores (mean ± SD)	t or *F* value	*P*
**Gender**				
Male	63	74.34 ± 14.00	-0.336	0.715
Female	64	73.51 ± 11.23		
**Birthplace**				
City	74	66.38 ± 9.15	-1.11	0.269
Countryside	53	64.55 ± 9.20		
**Frequency of reading literature**				
More than 4 articles per week	13	78.75 ± 14.36	15.811	**0.000**
2–3 articles per week	58	79.01 ± 10.90		
Less than 1 article per week	56	67.53 ± 11.11		
**Time of PBL self-directed learning**				
More than 6 h per week	18	80.34 ± 13.45	3.498	**0.018**
4–6 h per week	39	74.41 ± 11.86		
2–4 h per week	48	73.93 ± 12.52		
Less than 2 h per week	22	67.78 ± 11.43		
**PBL performance scores ranking**				
Top 30%	42	75.37 ± 12.84	3.998	**0.021**
Between top 30% and last 10%	68	74.99 ± 12.24		
Last 10%	17	66.05 ± 11.44		

### A significant positive correlation was observed between the influential factors and the clinical thinking ability of medical students in the PBL group

To further understand the relationship between the influential factors and clinical thinking ability, multiple linear regression analysis was used to observe whether there was a positive correlation. In this study, clinical thinking ability was taken as the dependent variable, while the frequency of reading medical literature, time spent on PBL self-directed learning, and PBL performance score ranking were taken as independent variables. As presented in Table 5, the results indicated that an increased frequency of reading medical literature and higher PBL performance score ranking were correlated with a higher clinical thinking ability. However, the time spent on PBL self-directed learning was not correlated with the clinical thinking ability score.

**Table 5:** Multiple linear regression analysis of influencing factors on clinical thinking ability of medical students for the PBL group

Factors	B	SE	Beta	t	*P*	95%CI
Constant term	49.155	4.782		10.278	**0.000**	39.782 ~ 58.528
Frequency of reading literature	6.811	1.634	0.354	4.170	**0.000**	3.610 ~ 10.013
Time of PBL self-directed learning	1.885	1.054	0.152	1.789	0.076	-0.180 ~ 3.95
PBL performance scores ranking	4.014	1.540	0.208	2.607	**0.010**	0.996 ~ 7.031

Explanatory note: B, Nonstandard regression coefficient; SE, Standard error; Beta, Normalized regression coefficient; F = 11.624, adjusted *R*^2^ = 0.202, *P* < 0.05

## Discussion

In the era of mass data and information explosion, cultivating students’ creative thinking has become an important task in education. PBL can contribute to the acquisitions of self-regulatory and reasoning skills, supporting the development of strategies for productive reasoning and creative thinking by solving instructional problems [13, 14]. Medical curricula aim to help students connect clinical with basic medical knowledge. However, traditional curricula struggle to integrate these two areas, making the application of PBL a potential solution to some of the problems existing in traditional curricula.

PBL has been described as an effective and efficient strategy to encourage students to improve analytical, problem-solving and collaboration skills [15, 16], making it well-suited to building critical thinking skills [17]. Numerous studies have shown a positive relationship between PBL and critical thinking in nursing education [9, 18–20].

Despite this, studies of the effects of PBL on clinical thinking of medical students remain limited. This study aimed to clarify the impact of PBL on clinical thinking ability based on classical clinical thinking assessment questionnaire from Delphi research. The results demonstrated that the integrated PBL curriculum significantly improved clinical thinking skills, including the critical thinking ability, systematic thinking ability and evidence-based thinking ability. Therefore, it can be concluded that the integrated PBL curriculum may be a more effective way of developing clinical thinking ability for undergraduate medical students. It was worth noting that in the process of this curriculum, the PBL mode was implemented as a main part to summarize questions using mind maps and discussing clinical problems with clinicians was also an important strategy. Therefore, we believed that not only the PBL mode but also the comprehensive curriculum design mode helped medical students to develop their clinical thinking ability.

For medical students prior to clinical practice, PBL offers an entry into a complex and real clinical situation. Small group discussions centered on a patient’s narratives with history, physical examination, and laboratory data compromise the data, summarized as the problem list, for the discussion to extract meaningful concepts leading to diagnosis and management [21]. This process is similar to clinical reasoning, which is a practical method of clinical thinking. It is a process of collecting cues, processing the information, coming to an understanding of a patient problem or situation, planning and implementing interventions, evaluating outcomes, and reflecting on and learning from the process [22]. Thus the constructive PBL model enables students to cultivate high-order thinking skills such as critical thinking, systematic thinking, and creative thinking [23].

The formation of clinical thinking not only requires medical students to learn basic medical theoretical knowledge in school, but also requires medical students to enter clinical practice and transform the knowledge into clinical practice. The results of this study also illustrated that several factors influence the clinical thinking of undergraduate medical students. Furthermore, the increased frequency of reading literature and PBL performance score ranking had a significant positive correlation with higher clinical thinking ability.

Reading medical literature is one of the crucial ways for medical students to acquire knowledge gradually. The formation of clinical thinking ability requires a broad medical knowledge base as a foundation [24]. Additionally, reading medical literature can help students understand the scientific discovery process, improve their ability to interpret experimental data, and cultivate their interest in scientific research [25]. PBL scores, which were assessed by tutors and students in each group, reflected students' performance in various aspects of learning, such as learning attitudes, data preparation, communication, critical thinking, and team cooperation. Therefore, reading medical literature and performing well in PBL curriculum play a positive role in improving the clinical thinking ability of medical students by expanding their field of vision, cultivating their mode of thinking, and stimulating their critical thinking skills.

Our curriculum practice has demonstrated that PBL has effectively transformed students' learning concepts from passive to active and lifelong learning. Active learning approaches offer many advantages over traditional instructional methods, including improved retention of information, conceptual understanding, analytical reasoning, and problem-solving skills. These skills are essential for the transfer and application of classroom-acquired knowledge to the clinical setting.

However, some limitations of this study cannot be overlooked. Firstly, the clinical thinking and critical thinking assessment scores were obtained through a questionnaire, which may introduce issues with the validity of self-reported data, although this method is commonly used in the literature. Therefore, future studies should consider using more multidimensional evaluation tools for clinical thinking. Secondly, the participants in this study were undergraduate medical students who had not yet entered clinical practice, and the duration of the study was relatively short. Clinical practice time is known to be one of the factors that can affect clinical thinking ability [26]. Future studies with larger sample sizes, longer duration, and consideration of additional influencing factors will be necessary to further demonstrate the impact of the integrated PBL curriculum on clinical thinking ability.

## Conclusions

In summary, this study demonstrates that an integrated PBL curriculum can help undergraduate medical students improve their clinical thinking ability. Furthermore, the study also identifies the influencing factors that are positively correlated with clinical thinking in undergraduate medical students prior to clinical practice. Although there is evidence that active learning approaches like PBL are effective in developing critical thinking, the findings are inconclusive, and clinical thinking is not exactly equivalent to critical thinking. Our research provides new evidence to show a positive influence of PBL on the clinical thinking of undergraduate medical students. Therefore, the PBL teaching strategies should be continuously carried out to improve the clinical thinking of medical students.

## Data Availability

The datasets during and/or analyzed during the current study are available from the corresponding author on reasonable request.

## Abbreviations

PBL: Problem-based learning
LBL: Lecture-based learning
